# A Study of Environmentally Friendly Menstrual Absorbents in the Context of Social Change for Adolescent Girls in Low- and Middle-Income Countries

**DOI:** 10.3390/ijerph18189766

**Published:** 2021-09-16

**Authors:** Jasmin Foster, Paul Montgomery

**Affiliations:** 1 Abbotsleigh School, Sydney, NSW 2076, Australia; 2 School of Social Policy, University of Birmingham, Birmingham B15 2TT, UK; p.x.montgomery@bham.ac.uk

**Keywords:** menstruation, adolescence, low income, sanitary pad, biodegradable, bamboo, gender equality, absorbency, menstrual hygiene

## Abstract

Girls in low- and middle-income countries struggle to maintain good menstrual hygiene in part due to a lack of affordable sanitary products. The unaffordability of reliable sanitary products can lead to school absenteeism and is a barrier to education attainment and gender equality for girls in low-income contexts. Further, the lack of adequate disposal facilities can lead to social embarrassment and environmental pollution. Low-cost biodegradable absorbents (cotton terry cloth, linen, hemp cloth and bamboo wadding) were investigated in gelatine solution in terms of their absorption for use in menstrual hygiene. Bamboo wadding exhibits the highest absorption index (7.86), greater than cotton terry cloth (0.84), hemp cloth (1.4), linen (1.57) and a commercial sanitary pad (4.38). Though the absorption index of bamboo wadding is promising, especially in light of the vast availability of bamboo in tropical low- and middle-income countries, challenges lie in overcoming complex extraction processes from bamboo plants, which is discussed. This simple research of a physical material from a technical aspect, if further investigated with a social science and policy lens, could increase school attendance, improve the education levels attained by girls and be a key step towards gender equality in low- and middle-income countries.

## 1. Introduction

Women in low- and middle-income countries struggle to maintain good menstrual hygiene. Most research to date details the challenges of menstrual hygiene management in sub-Saharan Africa and South Asia [1]. However, studies conducted in East Asia, Latin America, Caribbean and the Middle East also highlight consistent challenges in women’s menstrual experience [2]. These struggles are in part due to a lack of affordable sanitary products [3]. Poor menstrual hygiene can cause increased vulnerability to urinary and reproductive tract infections, which can lead to infertility and other reproductive organ issues [4,5]. Moreover, unaffordability of sanitary products restricts active participation of girls in school, contributing to gender inequality. For example, as many as 40% of girls in India are absent from school when menstruating [6] and in Africa it increases to 66% [7]. If sanitary products could be made affordable in terms of cost and product quality, then it will be possible to significantly increase school attendance, allowing girls to complete their basic education [8]. As widely reported in the literature, females staying longer in school is linked to reduced maternal death, improved population health, increased contraceptive uptake, improved child health, increased vaccination rates and decreased HIV infection rates [9,10].

The traditional ways of managing menstrual bleeding in such countries is the use of old clothes, paper, cotton, wool pieces, and even leaves which have unreliable levels of absorbency. These unreliable absorbents of traditional menstrual hygiene products can keep girls away from attending school. Therefore, provision of superior absorbents and cost-effective menstrual hygiene products can reduce fears of soiling outer garments allowing better school attendance [11]. Further, schools have insufficient private changing areas, poor water/sanitation, and inadequate disposal facilities. This results in disposal of menstrual hygiene products in deserted areas or in latrines to avoid embarrassment, causing environmental pollution [12]. Developments such as the re-usable menstrual cup require addressing significant concerns such as pain when inserting, anxiety of the cup “getting stuck” and concerns from relatives that the use of the menstrual cup leads to reduced fertility or “losing virginity” [13]. Another way to address this matter, while aligning with current habits is the use of fabric pads and/or disposable pads manufactured from biodegradable materials such as bamboo fibres, hyacinth and banana fibres. Having said that, commercial biodegradable products are not readily available and cost-effective which restricts broad penetration of these products into low- and middle-income communities [14]. Thus, an alternative approach is required that will address how commonly available fabrics of biodegradable natural fibres can be used as sanitary pads. This could help billions of women in low- and middle-income countries to improve menstrual hygiene management. Additionally, this paper is in line with the broader “MHM (Menstrual Hygiene Management) in Ten” 2014–2024 global agenda of providing girls with support in the school environment to manage menstruation with dignity, safety and comfort. Specifically, it responds to the recommendations to advance the agenda of ‘Priority 1: Build a strong cross-sectoral evidence base for MHM in schools for prioritization of policies, resource allocation, and programming at scale’. It could address the need for ‘natural experiments’ to understand the funding and policy implications of MHM programs in schools that provide menstrual products to girls in middle and low-income countries [15]. Further, this study, which provides new preliminary evidence in the area of health, efficacy, environmental safety of menstrual products, is in line with the global consensus regarding adolescent menstrual health in low- and middle-income countries and suggestions for future action and research [16].

The aim of this investigation is to analyse the absorption capacity of readily available, natural biodegradable materials for the purpose of feminine sanitary hygiene products in low- and middle-income countries. Together with that, strategies for using these natural biodegradable materials in a cost-effective way by involving local NGOs (Non-Governmental Organisations) were also discussed.

### Biodegradable Materials for Sanitary Pads

The most common material used for commercial sanitary pads is superabsorbent polymer (SAP). This material was first utilised for sanitary pad and diaper manufacture in high-income countries (Japan and the US) in the 1970s. The challenges regarding SAP are that it is expensive, and the production is more technical, requiring a high level of capital and complex machinery.

In contrast to SAP, natural plant fibres are cellulose-based and attract water which make them highly absorbent. The structure of plant fibres changes dimensions with changing moisture content because the cell wall contains hydroxyl and other oxygen containing groups that attract moisture through hydrogen bonding. Moisture swells the cell wall, and the fibre expands until the cell wall is saturated with water. Beyond this saturation point, moisture exists as free water in the void structure and does not contribute to further expansion. Superabsorbent polymer can absorb up to 200-fold of its own weight of water [17]. Cotton fibres, from cotton plants, typically hold water up to 24–27-fold their own weight [18].

Linen fibres, which are obtained from the flax plant, have less absorbency than cotton fibres [19]. Cotton terry cloth, where cotton fibres are woven in loops, is more absorbent than standard cotton. The surface area of the loops is designed to absorb liquids and the ability of absorption is driven by fabric weight, thickness, and pile yarn twist [20]. Hemp or industrial hemp is a natural fibre from a variety of the Cannabis sativa plant. Hemp has antibacterial properties and good absorbency [21,22]. Hemp is more water absorbent than cotton [23]. Bamboo fibre or bamboo textile is another highly absorbent material. Bamboo fibre is also more absorbent than cotton [24]. A study in cloth diapers, comparing bamboo diapers, cotton diapers and blended fabrics found that pure bamboo has the strongest antibacterial activity and a bamboo cotton blend had greater absorption capacity than pure cotton [25]. The cross-section of the bamboo fibre is filled with numerous micro-holes and micro-gaps. Bamboo fibres’ cellulose composition consists of crystalline and hierarchal structures which differs from the other natural materials. Bamboo is also found to contain a unique anti-bacterial and bacteriostasis bio-agent called ‘Bamboo Kun’. This feature of bamboo fibre makes it useful for sanitary products, as it will not gather as much bacteria as other alternatives, when worn for extended periods. Bamboo fibre appears to be an excellent alternative to SAPs, as it is highly absorbent, biodegradable and has excellent ventilation and several anti-bacterial properties. However, processing of bamboo fibre and sealing it into a sanitary pad is expensive, which in turn increases the user cost. In view of that, direct usage of bamboo wadding fabric instead of bamboo fibres was investigated in this current study. Bamboo wadding fabric has been used previously only inside quilts and children’s coats.

## 2. Materials and Methods

**Figure 1:**
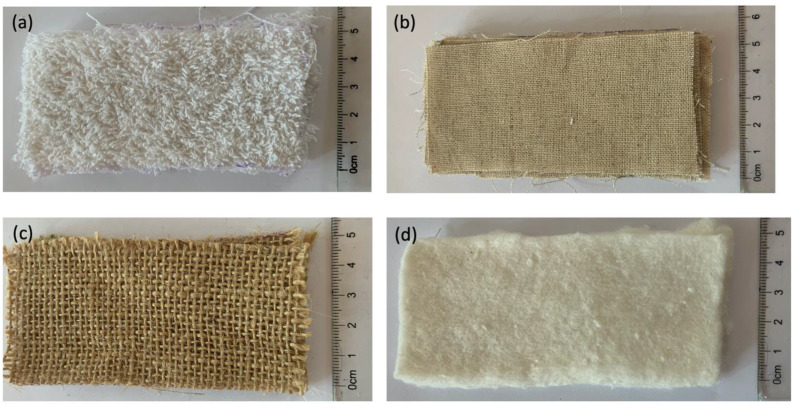
Optical photograph of the biodegradable materials used in the present study before absorption of gelatine solution; (**a**)—100% cotton terry cloth, (**b**)—100% linen cloth, (**c**)—100% hemp cloth, (**d**)—100% bamboo wadding.

Four different kinds of natural biodegradable material, namely (a) 100% cotton terry cloth, (b) 100% hemp cloth, (c) 100% bamboo wadding and (d) 100% linen, were investigated in this study to find out their respective absorption index. All the fabrics are commercially available in bulk off the shelf and procured accordingly. The fabrics were cut by hand to give them a rectangular shape and stacked to 1 cm thickness as shown in Figure 1. Together with that, the absorbency of a regular, store-bought sanitary pad (1 cm thick) was investigated as a comparator for the other materials. To ensure reproducibility of the results, all the experiments were triplicated. The data were recorded in July 2020 at the science laboratories of Abbotsleigh School.

A gelatine solution was used to imitate the viscosity of menstrual fluid to provide a more realistic experiment. The typical volume of menstrual fluid lost during a monthly menstrual cycle is approximately 10–80 mL over eight days (average menstrual cycle). In the present study, 20 mL of liquid (gelatine solution) was used to test the absorbency of these natural materials to ensure that the fabrics can retain more than one day’s worth of menstrual fluid. At first, 10 g of gelatine was added into 300 mL of water and heated (60 °C) with stirring until there were no remaining gelatine particles visible. The solution was then split up into 15 × 20 mL volumes for each fabric. The temperature of each solution was then checked to ensure the liquid was at room temperature (21 °C) to maintain a constant gelatine solution viscosity, visually representative of menstrual fluid that could remain across each test fabric. Then, each of the test fabrics was weighed on a precision lab-scale, placed into the container of the same size and 20 mL of the gelatine solution was poured. After 60 s, the fabric was removed from the container and weighed again. The absorption index (ratio of absorbed mass to dry weight) was calculated. An absorption index was used to ensure that the results could be comparable, as each fabric has a different dry weight. Parallax and reading errors were reduced by setting each measuring cylinder down on a flat surface and measuring the gelatine liquid from eye-level. Random cross checks were conducted by a lab assistant.

## 3. Results

Table 1 shows the relevant data together with respective standard deviation from statistical analysis. As seen in Table 1, cotton terry cloth had the lowest average absorption index (0.84 ± 0.15), even than that of linen (1.57 ± 0.16) and hemp (1.40 ± 0.17), which is contradictory with respect to the research reported in the literature [19,26]. Bamboo wadding had the highest average absorption index (7.86 ± 1.01), followed by the Kimberly-Clark Kotex (sold in Australia as the Kotex Super Ultrathin with Wings) commercial sanitary pad (4.38 ± 0.02). The absorption index of bamboo wadding was almost twice that of the commercial sanitary pad.

Bamboo wadding had the greatest mass of absorbed solution (19.69 ± 0.06), which was similar to the mass of absorbed solution of the Kotex commercial sanitary pad (19.49 ± 0.21). However, the sanitary pad was 1.8-fold the starting dry weight of the 1 cm stacked bamboo wadding.

Cotton terry cloth had the lowest mass of absorbed solution (6.67 ± 1.23), followed by hemp cloth (7.86 ± 0.97) and linen (10.26 ± 0.44).

## 4. Discussion

According to [27], bamboo fibre is more absorbent than cotton. However, this experiment revealed that bamboo fibre in a non-woven wadding form was 9-fold more absorbent than cotton, and almost twice as absorbent as a standard sanitary pad.

The superior absorption of the bamboo wadding is due to the unique structure of its fibre. Bamboo fibres are composed of a different type of cellulose structure, which differs from that of the other materials. While all cellulose sugar molecules can break a liquid’s surface tension and allow the liquid to absorb into spaces between fibres, and into fibres themselves, bamboo cellulose’s crystalline and hierarchal structure differs, making the fabric more absorbent. Bamboo has good overall moisture management capability, which classifies the material as water penetration fabrics with small spreading area [28]. A recent study found that bamboo fibre as an absorbent core in a traditional sanitary napkin format absorbs and wicks water 3–4-fold better than cotton and reduces odour as the fibre is filled with multiple micro-holes and micro-gaps [29]. Thus, the use of bamboo fibres as a core of sanitary pads is a good alternative compared to SAP and, moreover, it is biodegradable in nature. In addition, bamboo fibre in wadding form, as investigated in the present study, is more convenient to use, and lower in cost in comparison with bamboo fibre embedded commercial sanitary pads. For example, off the shelf, bamboo wadding is approximately US $0.50–US $2.00 per square metre (https://www.alibaba.com/product-detail/bamboo-batting-for-baby-quilts-bamboo_62008249222.html?spm=a2700.7724857.normalList.122.279169e7PTKY2h (accessed on 20 July 2020)); and each square metre could make up to 40 sanitary pads. Thus, each of the sanitary pads cost approximately US $0.0125–US $0.05. For bamboo wadding to be adopted for use as a sanitary pad there are issues to be further examined around its potential scale up and production which may hamper a broad uptake. Bamboo is widely available in tropical/sub-tropical countries with global economy value over $60 billion per year [30,31] The process of extracting fibres from bamboo is complex and requires substantial investment and expertise. (The complexity of production was discussed with the owners of Australian manufacturer Victorian Textiles. https://www.victoriantextiles.com.au/M950_dash_240/100%25-Bamboo-2.4m-x-30m-Roll/pd.php (accessed on 15 October 2020)) After China, India is the second largest producer of bamboo; however, the manufacture of bamboo fabric is underdeveloped. There are two main methods of producing bamboo fibres, namely mechanical and chemical. The mechanical method has been found to be more eco-friendly (though more expensive) as it does not use or create chemicals. The fibre extracted by mechanical process is where the bamboo culm (jointed stem) is split mechanically followed by rasping off the woody part. The crushed bamboo strands are treated with enzymes to separate the fibrous materials from the remaining stem. The individual fibres are then combed out and spun into yarns. The chemical process is where the bamboo culm is crushed into smaller fractions and soaked in a solution of 18% sodium hydroxide (NaOH) at 20–25 °C for 1–3 h to form alkali cellulose. The bamboo alkali cellulose is pressed to remove excess NaOH solution, crushed by a grinder and left to dry for 24 h. In this stage, carbon disulfide (CS_2_) is added to the bamboo alkali cellulose to sulfurize the compound, causing it to gel. The remaining CS_2_ is removed by evaporation due to decompression. A diluted solution of NaOH is added to the cellulose sodium xanthogenate, which dissolves it into a viscose solution consisting of approximately 5% NaOH and 7–15% bamboo fibre cellulose. The viscose solution is forced through spinneret nozzles into a larger container of diluted sulfuric acid (H_2_SO_4_) solution, which hardens the viscose and reconverts it to cellulose bamboo fibres which are spun into yarns and given the shape of bamboo wadding [32]. For sanitary pad production, the bamboo fibres are then covered in a polythene and non-woven sheet and released for cutting. The hygienic napkins are then sealed with adhesive and perfumed before sending for packing. However, this approach became commercially unviable in practice due to the complexity of the process as well as initial investment [33,34]. Having said that, an alternative of large-scale commercialization is the small-scale handicraft-based approach involving the people who are going to use it. In this approach, non-governmental organizations (NGOs) may take the lead, as NGOs in low- and middle-income countries are playing a significant role in socio-economic developments. For example, Goonj [35], an Indian NGO in New Delhi, currently collect urban surplus fabrics, then wash, dry and cut them into pads which are packed and distributed via partner grassroots NGOs. It is possible to involve such NGOs to distribute low-cost bamboo wadding to replace less absorbent surplus fabrics, with users hand cutting the fabric to give the required shape. It is also possible to involve such NGOs to train-up local communities to make bamboo wadding from plants. This will reduce the cost, make it readily available among school girls and women and could have a significant transformative effect. Moreover, bamboo wadding is re-usable in nature.

Future investigations should include in-use testing to assess the safety of non-woven bamboo and the chemicals used in the manufacturing process. This process for making batting for the inside of quilts has been used in the USA since 1936 but have not been tested for use as a menstrual absorbent. In-use testing would also assess the ability of the product to hold fluid under pressure, the ability to withstand washing and absorbency over time.

## 5. Conclusions

In the present research, the absorption index of a number of biodegradable materials was investigated with gelatine solution and compared against a commercial sanitary pad. It was found that bamboo wadding was the most absorbent natural material in comparison to hemp cloth, linen and cotton terry cloth. Being nearly twice as absorbent as a commercial sanitary pad, bamboo wadding appears to be the most suitable material for the use of sanitary products as it is extremely absorbent, affordable, lightweight, biodegradable, has no detrimental effects on the user or the environment. This experiment is exploratory and requires further replication and investigation but is a promising start in this field. Further research could enable girls in low- and middle-income countries to make their own sanitary pads of a quality superior to those in high-income countries from bamboo plants in their villages. This research of a physical material from a technical aspect, if further investigated with a social science and policy perspective, could increase school attendance, improve the education levels attained by girls and be a key step towards gender equality.

## Figures and Tables

**Table 1:** Absorption index of the materials investigated in the present study.

Material	Dry Weight (g)	Wet Weight (g)	Mass of Absorbed Solution (g)	Absorption Index
Kotex sanitary pad	4.45 ± 0.06	23.94 ± 0.27	19.49 ± 0.21	4.38 ± 0.02
Cotton terry cloth	7.93 ± 0.03	14.61 ± 1.23	6.67 ± 1.23	0.84 ± 0.15
Hemp cloth	5.75 ± 0.10	13.61 ± 0.96	7.86 ± 0.97	1.40 ±0.17
Linen	6.55 ± 0.43	16.81 ± 0.10	10.26 ± 0.44	1.57 ± 0.16
Bamboo wadding	2.50 ± 0.34	22.23 ± 0.30	19.69 ± 0.06	7.86 ± 1.01

g—grams.

## Data Availability

The datasets used and/or analysed during the current study are available from https://doi.org/10.6084/m9.figshare.14349404.v1 (posted on 1 April 2021, accessed on 21 July 2021).
